# Combined effects of species diversity and soil depth heterogeneity on plant functional groups and community productivity

**DOI:** 10.7717/peerj.21225

**Published:** 2026-05-01

**Authors:** Lu Liu, Bohui Cheng

**Affiliations:** 1 College of Water Resource, North China University of Water Resources and Electric Power, Zhengzhou, Henan Province, China; 2 School of Design, Henan Mechanical and Electrical Vocational College, Zhengzhou, Henan Province, China

**Keywords:** Soil spatial heterogeneity, Soil depth, Species diversity, Community productivity, Selection effect, Plant functional groups

## Abstract

Fine-scale heterogeneity in soil nutrient availability can enhance plant growth, yet its effect across soil depth remains inadequately understood. Communities with increased species diversity showed varying growth strategies among different plant functional groups to occupy soil niches. However, the interactive effects of soil depth heterogeneity and species diversity on plant functional groups and community productivity remain poorly known. This study aimed to examine whether plant functional groups respond differently to soil depth heterogeneity, whether such heterogeneity modifies interspecific relationships and community productivity, and whether higher species diversity alters plant growth strategies under vertical resource variation. A greenhouse experiment was conducted using twelve common herbaceous species with diverse functional traits and root strategies grown under one homogeneous and two heterogeneous treatments that varied in patch size, crossed with two species diversity levels. Plant species differed in responses to soil depth heterogeneity. Increased soil depth promoted the growth of legumes and forbs and enhanced community productivity. In high-diversity mixtures, forbs accumulated greater aboveground biomass in deeper heterogeneous soils, whereas grasses showed no significant response to soil depth or diversity. Legumes displayed a flexible growth pattern, reducing allocation to deeper layers, which may limit competitive pressure. Higher species diversity strengthened the selection effect in forbs, increasing their proportional contribution and intensifying interspecific competition under soil depth heterogeneity. This study provides experimental evidence for soil–plant interactions driving species coexistence and community productivity. Decreased-scale heterogeneity may also function as a reduced-scale form of habitat fragmentation. Long-term field experiments and multi-dimensional niche analyses are needed to better understand community dynamics across gradients of biotic and abiotic factors.

## Introduction

In natural ecosystems, soil properties often manifest as patchiness, where resource levels are relatively uniform within patches but vary significantly between them (Hutchings & John, 2004; Staudacher et al., 2018). Such spatial soil heterogeneity is common and frequently occurs on fine scales relevant to plant growth (Liu et al., 2017a; Xue, Huang & Yu, 2021). Fine-scale heterogeneity in soil nutrient availability can increase plant growth when the scale of individuals exceeds the scale of nutrient patches (Hendriks et al., 2015). This positive effect of heterogeneity is likely attributable to plants concentrating roots where resource availability is relatively high (García-Palacios et al., 2012; Kembel & Cahill, 2005), a response known as root foraging (de Kroon et al., 2009). The species differ in root foraging ability, and those with greater capacity tend to show greater positive effects of soil heterogeneity on growth (Bliss et al., 2002; Xue, Berendse & Bezemer, 2018).

Previous studies have examined plant species’ responses to soil heterogeneity, primarily focusing on nutrient or water availability across spatial depths (Baer et al., 2019; Ruijven & Berendse, 2010; Stevens & Jones, 2006). When soil depth is limited, essential growth resources such as nutrients and water decline linearly with spatial constraints, leading to intensified competition or altered growth strategies (Hu et al., 2024; Krüger, Finn & Don, 2024). Experiments in temperate grasslands have demonstrated that deeper soils support greater productivity in plant communities, with the effects of soil depth on biomass qualitatively resembling those of fertilization (Braun et al., 2022). Soil depth heterogeneity involves vertical stratification and uneven distribution of soil resources, influencing physical, chemical, and biological properties across depths and shaping resource availability for plants (Liu et al., 2017b; Teste & Laliberté, 2019). This may drive plant functional groups to allocate resources *via* root system distribution across depths, enhancing exclusive resource use at different levels and representing a primary form of niche differentiation (Martorell et al., 2015; Yang et al., 2023).

The primary consequence of niche differentiation is the expansion of available niches. Greater soil depth heterogeneity provides additional vertical resource niches, which can support a wider range of plant functional groups and promote species diversity (He et al., 2025; Schenk, 2010). In turn, increased species diversity within a community enhances its sensitivity to soil depth heterogeneity. This bidirectional interaction strongly influences plant diversity *via* functional group richness, uniformity, and complementarity, shaping both community structure and productivity (Costanza, Moody & Peet, 2011; Maestre & Reynolds, 2006). In mountain ecosystems, soil layers often vary in thickness, enabling the coexistence of deep-rooted trees, shallow-rooted shrubs, surface herbs, and nitrogen-fixing legumes through increased niche availability. This results in higher functional group richness compared to homogeneous plains (Zheng et al., 2025).

Soil depth heterogeneity also affects the intensity and direction of interactions among plant functional groups, potentially transforming competitive relationships into coexistence with mutual benefits (Van Steijn et al., 2025). Enhanced soil depth heterogeneity can direct distinct functional groups to occupy specific ecological niches across different layers, reducing interspecific competition for shared resources; further facilitate mutualistic interactions, as shallow-rooted nitrogen-fixing plants improve soil nutrient availability for neighboring deep-rooted species, creating mutually beneficial associations; moreover, limit the dominance of competitive functional groups that monopolize resources under uniform soil conditions, allowing less competitive groups to establish (Brooker et al., 2008; Hou et al., 2023; Silvertown, 2004; Tilman, 1994). In uniform farmland soil, weeds are often dominated by shallow-rooted species, whereas in naturally heterogeneous soils, deep-rooted weeds can coexist with shallow-rooted crops by exploiting deeper resources (Baer et al., 2019).

Various studies have emphasized the roles of complementarity and selection effect in driving biodiversity and community productivity (Loreau & Hector, 2001; Oram et al., 2018; Wang et al., 2024). Communities with higher richness typically display greater productivity, as indicated by increased aboveground biomass (Gracia et al., 2025). The complementarity effect arises when diverse species occupy distinct ecological niches, enhancing resource utilization within the community and increasing total biomass (Daleo et al., 2023; Šímová, Li & Storch, 2013; Xue, Huang & Yu, 2021). The selection effect suggests that high-diversity communities are more likely to harbor certain “super species” with high yield, which confer advantages in mixed planting and elevate overall productivity (Ron, Fragman-Sapir & Kadmon, 2018). These effects are particularly evident under patchy soil conditions (Šímová, Li & Storch, 2013). However, current research remains limited, with few studies directly linking the influence of soil depth on productivity to changes in species diversity (Braun et al., 2022; Liu et al., 2020b).

To address this gap, twelve common herbaceous species with diverse growth rates, functional traits, and root strategies were selected to establish a local community. A greenhouse experiment was conducted to examine the effects of soil spatial heterogeneity and species diversity on plant community productivity, testing the following hypotheses: (1) plant functional groups show distinct responses to soil depth heterogeneity, linked to variations in their foraging behavior and root traits; (2) soil depth heterogeneity alters interspecific relationships among functional groups and influences community productivity; (3) higher species diversity within a community increases sensitivity to soil depth heterogeneity, leading to divergent plant growth strategies.

## Materials and methods

### Study species

Twelve common northern plant species were selected to construct a native herbaceous community for this experiment. These included four Poaceae species: *Bromus inermis* Leyss., *Cynodon dactylon* (Linn.) Pers., *Lolium perenne* L. and *Poa annua* L.; four Leguminosae species: *Astragalus adsurgens* Pall., *Astragalus sinicus* L., *Medicago sativa* L. and *Trifolium repens* L.; two Asteraceae species: *Cichorium intybus* L. and *Sonchus oleraceus* L.; one Oxalidaceae species: *Oxalis corniculata* L. and one Plantaginaceae species: *Plantago asiatica* L. (see Table 1 for details). These species naturally coexist in northern China and reproduce primarily through seeds. For this study, the twelve species were grouped into three functional groups, each containing four species. All seeds were non-hybrid and obtained from a commercial supplier affiliated with the Chinese Academy of Agricultural Sciences, Beijing.

**Table 1:** Species information in the experiment. These species are four Poaceae species, four Leguminosae species, two Asteraceae species, one Oxalidaceae species and one Plantaginaceae species. These species naturally coexist in northern China and reproduce primarily through seeds. For this study, the twelve species were grouped into three functional groups, each containing four species. Data are from the eFlora of China: http://www.efloras.org.

Species	Family	Life history	Root traits
*Astragalus laxmannii* Jacq.	Leguminosae	Perennial	Thick tap roots
*Astragalus sinicus* L.	Leguminosae	Biennial	Deep tap roots
*Bromus inermis* Leyss.	Poaceae	Perennial	Rhizome, fibrous roots
*Cichorium intybus* I.	Asteraceae	Perennial	Deep, fleshy roots
*Cynodon dactylon* (L.) Persoon	Poaceae	Perennial	Rhizome, fibrous roots
*Lolium perenne* L.	Poaceae	Perennial	Fibrous roots
*Medicago sativa* L.	Leguminosae	Perennial	Deep and thick roots
*Oxalis corniculata* L.	Oxalidaceae	Perennial	Rhizome, fibrous roots
*Plantago asiatica* L.	Plantaginaceae	Perennial	Shallow, fibrous roots
*Poa annua* L.	Poaceae	Annual	Fibrous roots
*Sonchus oleraceus* L.	Asteraceae	Annual	Fibrous roots
*Trifolium repens* L.	Leguminosae	Perennial	Fibrous roots

### Experimental design

**Figure 1:**
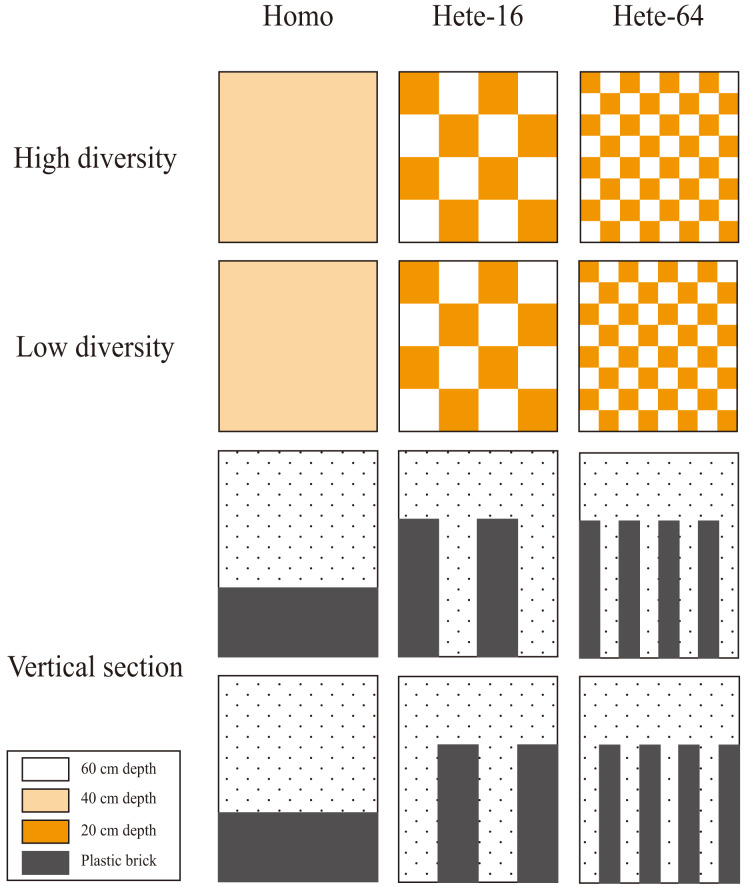
Experiment design. The experiment employed a fully factorial design with three soil treatments (large patch size heterogeneous *vs*. small patch size heterogeneous *vs*. homogeneous) and two species diversity levels treatments (high *vs*. low). Heterogeneous treatments that varied in patch size (12.5 *vs*. 6.25 cm). Each of six treatments combinations was replicated eight times, yielding 48 containers in total. Containers were constructed from polypropylene plastic and measured 50 cm × 50 cm × 60 cm (length × width × height). In heterogeneous treatments, containers included both shallow (20 cm) and deep (60 cm) soil patches at the designated scale levels, while the homogeneous treatment maintained the uniform medium depth (40 cm). The total volume was thus the same in all three soil treatments, approximately 100 liters.

The experiment employed a fully factorial design with three soil treatments (large patch size heterogeneous *vs*. small patch size heterogeneous *vs*. homogeneous) and two species diversity levels treatments (high *vs*. low). Heterogeneous treatments that varied in patch size (see Fig. 1 for details). Patch scale was the key determinants of soil heterogeneity, with representing the spatial extent over which depth changes occur. The larger size was intended to more closely match the rooting volume of individual plants and was thus expected to support greater productivity than the smaller size (Roiloa & Retuerto, 2006; Tamme et al., 2010). Each of six treatments combinations was replicated eight times, yielding 48 containers in total. Containers were constructed from polypropylene plastic and measured 50 cm × 50 cm × 60 cm (length × width × height).

In the large patch heterogeneous depth treatment, each container was conceptually divided into 16 equal patches (12.5 cm × 12.5 cm). Half of these patches were assigned either shallow (20 cm) or deep (60 cm) soil depths, referred to as Hete-16. This partitioning was arranged horizontally, ensuring that nutrient exchange between patches was not impeded. Plants were able to extend their roots across patches to access resources. To create shallow soil patch (20 cm), eight polystyrene bricks (12.5 cm × 12.5 cm × 40 cm) were placed at the bottom of alternating patches and secured with non-toxic aquarium silicone. In the small patch heterogeneous depth treatment, each container was divided into 64 equal patches (6.25 cm × 6.25 cm), and half of these patches were filled with 32 smaller polystyrene bricks (6.25 cm × 6.25 cm × 40 cm) to manipulate soil depth in the same manner as the large patch treatment. This arrangement is referred to as Hete-64. In the homogeneous depth treatment, each container was filled with a single larger polystyrene brick (50 cm × 50 cm × 20 cm) to maintain a standardized medium soil depth of 40 cm, referred to as Homo. The total volume was thus the same in all three soil treatments, approximately 100 liters. The growth substrate consisted of sand, peat-based substrate (Pindstrup Seeding, Pindstrup Mosebrug A/S, Ryomgaard, Denmark) and vermiculite mixed in a 1:1:1 volume ratio, supplemented with 2 g/L slow-release fertilizer (16N-9P-12K-2Mg; Osmocote 313S, Scotts, Marysville, Ohio, USA) to provide consistent nutrient availability. In the heterogeneous treatments, containers included both shallow (20 cm) and deep (60 cm) soil patches at the designated scale levels, while the homogeneous treatment maintained the uniform medium depth (40 cm). Effective soil depth in all treatments was measured using a custom probe to ensure proper placement of the polystyrene bricks. Deviations from the intended depths were ≦1 cm, likely due to slight compression of the bricks under soil weight.

In the high diversity treatment, the experimental plant community received 768 seeds from twelve species across three functional groups (64 seeds per species). In the low diversity treatment, the community consisted of 768 seeds from six species, representing half the number of species used in the high diversity treatment (128 seeds per species). Six species were randomly selected from twelve species such that each functional group contributed two species from four candidates without repetition, yielding 196 possible combinations in total. Eight combinations were then randomly selected from these 196 combinations and randomly assigned to repetitions (species combinations in the low diversity treatment are presented in Table 2). Across replicates, initial community composition in the high-diversity treatment was consistent, whereas composition in the low-diversity treatment differed among repetitions. Richness and sowing density were the same in two diversity treatment, whereas species composition were not.

**Table 2:** Species combination in low diversity treatment. Each combination comprises two grass species, two legume species and two forb species, randomly selected from twelve herbaceous species. Richness and sowing density were the same in two diversity treatment, whereas species composition were not. The total number of seeds planted was identical across all treatments, with 768 seeds per container, corresponding to a sowing density of 3,072 seeds per square meter.

Number	Species combination
1	*C. dactylon*, *L. perenne*, *T. repens*, *A. laxmannii*, *C. intybus*, *P. asiatica*
2	*L. perenne*, *B. inermis*, *A. laxmannii*, *M. sativa*, *P. asiatica*, *O. corniculata*
3	*P. annua*, *B. inermis*, *T. repens*, *M. sativa*, *S. oleraceus*, *P. asiatica*
4	*P. annua*, *L. perenne*, *T. repens*, *A. laxmannii*, *C. intybus*, *P. asiatica*
5	*P. annua*, *L. perenne*, *T. repens*, *A. sinicus*, *S. oleraceus*, *C. intybus*
6	*C. dactylon*, *B. inermis*, *A. sinicus*, *M. sativa*, *C. intybus*, *O. corniculata*
7	*C. dactylon*, *B. inermis*, *A. laxmannii*, *A. sinicus*, *S. oleraceus*, *O. corniculata*
8	*C. dactylon*, *P. annua*, *A. sinicus*, *M. sativa*, *S. oleraceus, O. corniculata*

Seeds were sown on 27 May 2022. In the Hete-16 treatment, each patch within eight containers assigned to the high-diversity treatment received four seeds per species, whereas each patch within another eight containers assigned to the low-diversity treatment received eight seeds per species. In the Hete-64 treatment, half of containers were sown with one seed per species per patch for the high diversity treatment, and the remaining containers received two seeds per species per patch for the low diversity treatment. In the Homo treatment, each container was sown following the same procedure as in the Hete-16 treatment. The total number of seeds planted was identical across all treatments, with 768 seeds per container, corresponding to a sowing density of 3,072 seeds per square meter. Monoculture experiments ensure equal total density for all plant species within the same treatment, which can be more suitable for assessing species responses to soil depth heterogeneity and interspecific competition. A meta-analysis of 452 experiments indicate that productivity increases on average by 15.2% from monocultures to species mixtures (Chen, Xiao & Chen, 2025). Monoculture treatments were not included in this experiment because the focus was on investigating the combined effects of soil depth heterogeneity and diversity on plant functional groups response and community productivity. The species showed differences in seed germination rates, resulting in varied community compositions at both the patch and container levels, despite identical initial sowing densities within each treatment. Sowing under these conditions more closely approximates natural environments, where diverse plant species experience environmental heterogeneity throughout their life cycles, starting from the seed stage.

The experiment concluded on 7 October 2022, after 19 weeks in a greenhouse with controlled 24-h constant temperature and humidity systems at North China University of Water Resources and Electric Power, Zhengzhou, China. The mean temperature and relative humidity in the greenhouse during the experiment were 25.4 ± 0.3 °C and 64.4 ± 0.8%, respectively, as measured by iButtons (DS1923; Maxim Integrated Products, Sunnyvale, CA, USA). Tap water was applied every 3 days to maintain soil moisture between 20–25%, a range higher than that of natural grassland but lower than that typical farmland. A randomized block design was implemented, with each repetition assigned to a block, ensuring that community composition was nested within blocks for statistical analysis. Each block contained one replicate of each heterogeneity by diversity treatment, and treatments within each block were randomly arranged. All containers were positioned in a two-dimensional array (8 × 6, latitudinal × longitudinal) in the greenhouse. Container placement was randomly changed every 4 weeks, totaling four rotations during the experiment. Although long-term community experiments provide stronger evidence, in this study, most plants had reached anthesis and fruiting before October, completing a full growing season from germination to reproduction.

### Measurement and data analysis

As harvest, all plants in each patch were cut at the soil surface and sorted into three functional groups (grasses, legumes and forbs). Roots were then harvested collectively from the central regions (25 cm × 25 cm) of each container. Roots were collected from two shallow and two deep patches in Hete-16 treatment; from four shallow and four deep patches in Hete-64 treatment; from four medium patches in Homo treatment. Roots were sampled from the central regions because these individuals were located within the interior of the community and experienced intensified interspecific competition, with roots able to extend into neighboring patches. Harvested roots were hand-washed using a 2-mm sieve. Due to the high intermixing of roots within each patch, they were pooled as a mixture. All plant materials were dried at 70 °C for 72 h and weighed.

Three-way, repeated-measure Analysis of Variance (ANOVA) were used to evaluate the effects of soil heterogeneity (heterogeneous or homogeneous), diversity (high or low), and soil patch (shallow or deep, repeated measure) on total aboveground biomass, aboveground mass of grasses, aboveground mass of legumes, aboveground mass of forbs, and root mass. Heterogeneity and diversity were treated as fixed effects, block (*i.e.*, species composition) as random effect, and soil patch as a repeated measure. As indicated by the experimental design diagram (see Fig. 1), soil depth in the homogeneous treatment was 40 cm, whereas in the heterogeneous treatment it was 20 and 60 cm, without physical barriers between them. Therefore, the soil patch was a non-independent within-subjects variable nested within heterogeneity. In the heterogeneous treatment, the soil patch corresponded to shallow or deep patches (20 or 60 cm). In the homogeneous treatment, soil depth did not differ but was compared for purposes of analysis to shallow and deep patches in the heterogeneous treatment based on spatial locations. For example, patches specified in the homogeneous and heterogeneous treatments with the same diversity treatments within the same replicate were compared to each other. Differences between individual means were tested with linear contrasts based on ANOVA. Analyses were conducted using SPSS 22.0 (IBM Corp., Armonk, NY, USA). Statistical significance was defined as *p* < 0.05. To quantify the effect of soil heterogeneity on community productivity between Hete-16 and Hete-64 treatment, the effect at two scales was calculated as: (mean total aboveground biomass in the heterogeneous treatment with low- or high-diversity minus mean total aboveground biomass in the homogeneous treatment with low- or high-diversity) divided by mean total aboveground biomass in the latter treatment. The distribution of such effect is symmetrical around zero, with negative values indicating inhibition and positive values indicating facilitation.

## Result

**Figure 2:**
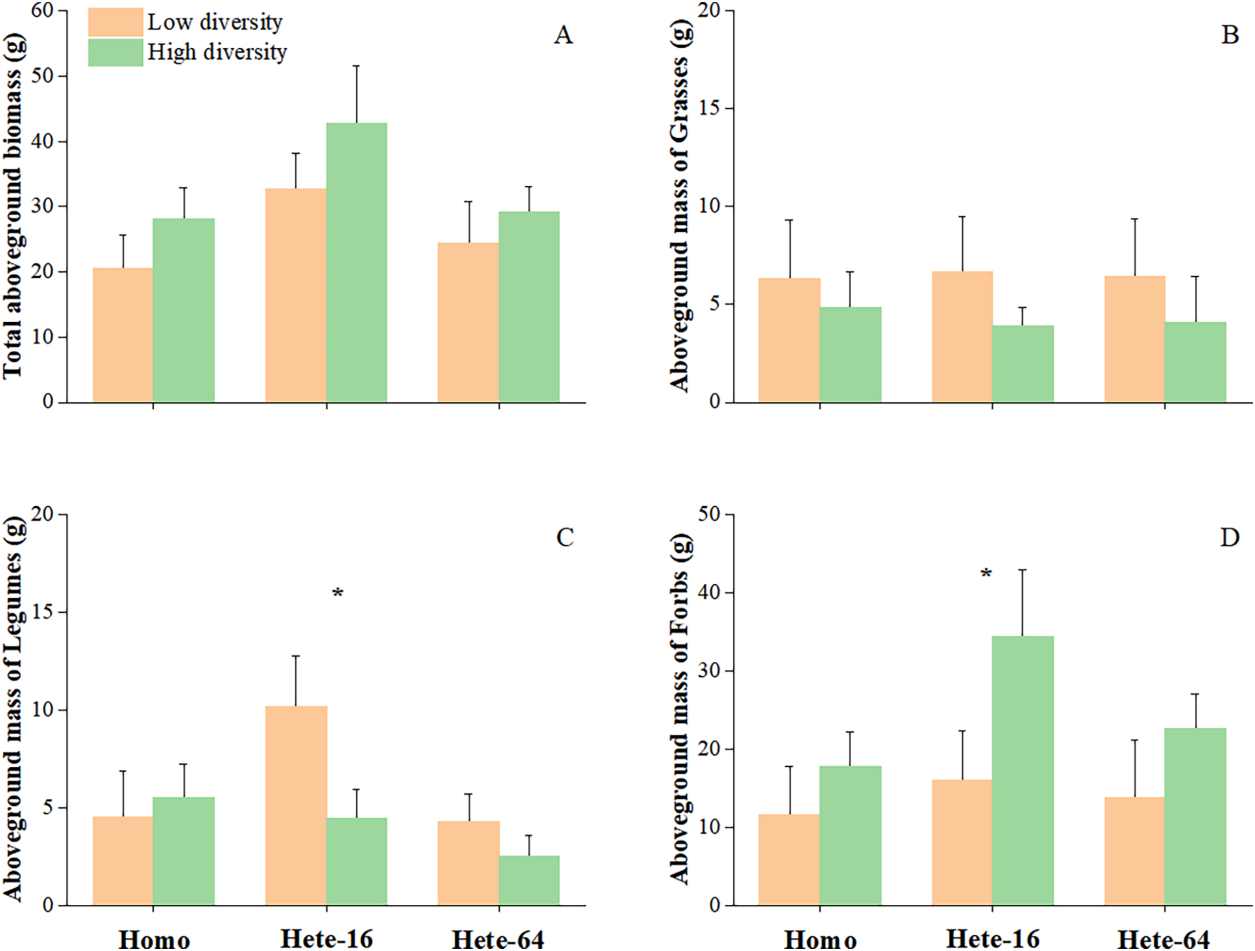
Effects of soil heterogeneity and species diversity on total aboveground biomass of community (A), aboveground mass of grasses (B), legumes (C) and forbs (D). The error bars are expressed as mean + SE. Symbols above pairs of bars show *P* (linear contrast based on ANOVA): none - >0.1; *0.01–0.05.

Across all experimental treatments, block (species composition) significantly influenced aboveground growth of all plants, therefore affecting overall community biomass accumulation (Table 3). Species diversity primarily impacted the total aboveground biomass and the aboveground mass of forbs, with higher species diversity associated with greater accumulation in forbs, and the entire community (Figs. 2A and 2D). Diversity also had a marginal effect on aboveground mass of legumes, indicating that legumes in the low diversity treatment tended to accumulate relatively more aboveground mass (Fig. 2C). Grass showed no response to the diversity treatment and did not differ in aboveground mass accumulation (Fig. 2B). Statistical analyses of variance are summarized in Table 3.

**Table 3:** ANOVAs of effects of block (B), species diversity (D), soil heterogeneity (H) and soil depth patch (P) on aboveground mass of community, grasses, legumes and forbs. Diversity and heterogeneity are treated as fixed effects, block (*i.e*., community composition) as random effect, and soil depth patch as a repeated measure.

Effects	*df*	Aboveground biomass	Aboveground mass of grasses	Aboveground mass of legumes	Aboveground mass of forbs
Bewteen-subjects:					
Block	7	4.76***	4.99***	3.01**	5.78***
Diversity (D)	1	4.0*	2.0	3.38^#^	9.14**
Heterogeneity (H)	2	4.24*	0.11	4.49*	2.9^#^
D × H	2	0.1	0.11	3.7*	0.79
Within-subjects:					
Patch (P)	1	0.91	0.11	0.01	0.69
P × D	1	0.01	0.04	2.93^#^	0.2
P × H	2	0.26	0.22	0.01	0.26
P × D × H	2	0.07	0.02	1.39	0.06
Error	77	——	——	——	——

**Notes**:

Symbols give *P*: none - >0.1.

# 0.05–0.1.

* 0.01–0.05.

** 0.001–0.01.

*** <0.001.

Soil depth heterogeneity significantly affected total community biomass. Communities in Hete-16 treatment produced more aboveground biomass than those in other soil treatments (Fig. 2A), with the positive effect being strongest under high diversity conditions. Plant functional groups exhibited distinct responses to soil depth heterogeneity. Legumes accumulated more aboveground mass in Hete-16 treatment and less in Hete-64 treatment compared to homogeneous treatment (Fig. 2C). Forbs tended to accumulate more aboveground mass in Hete-16 treatment than in other soil treatments (Fig. 2D). Soil patch depth did not independently influence overall growth (Table 3).

**Figure 3:**
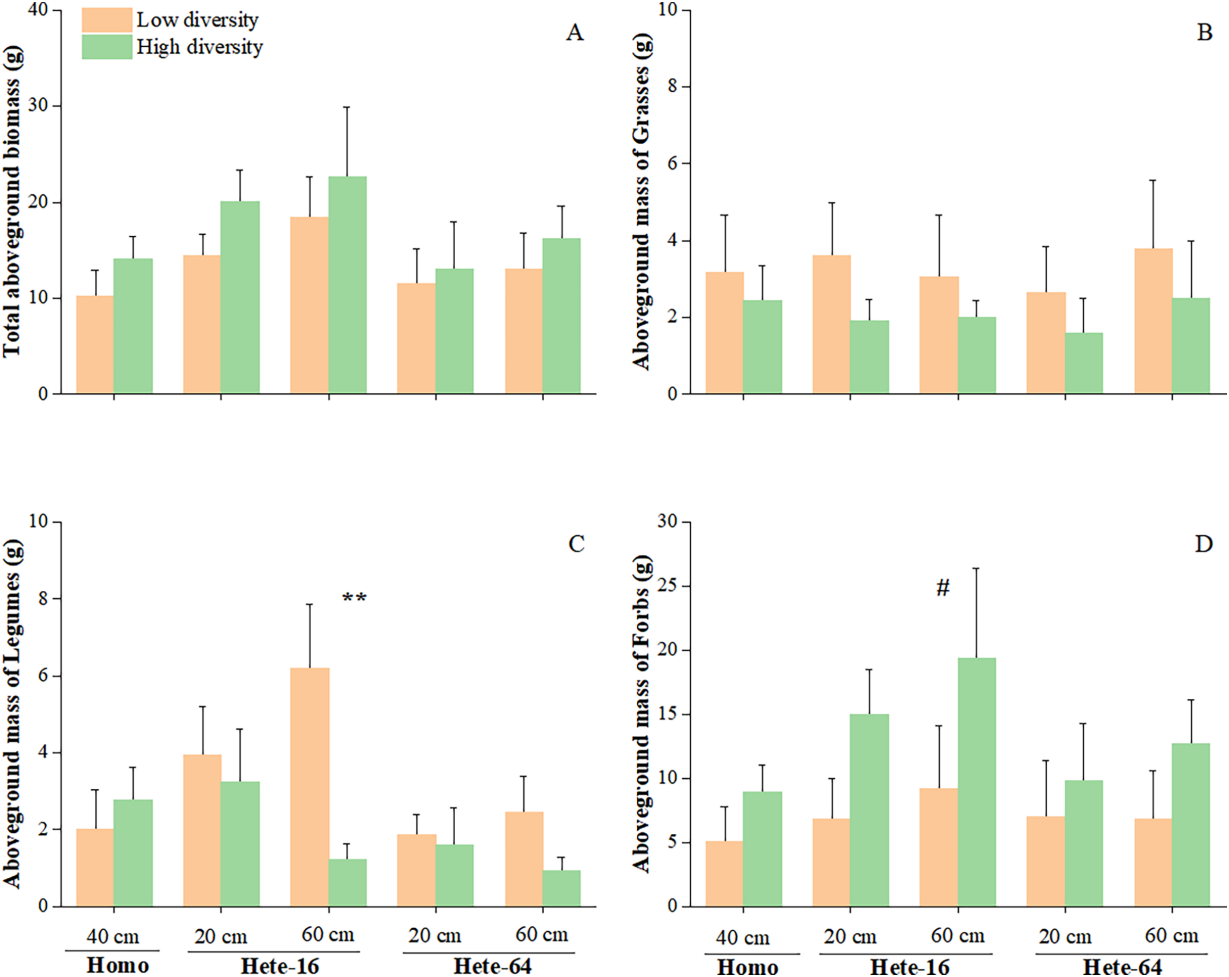
Effects of soil heterogeneity, species diversity and depth patch on total aboveground biomass of community (A), aboveground mass of grasses (B), legumes (C) and forbs (D). The error bars are expressed as mean + SE. Symbols above pairs of bars show *P* (linear contrast based on ANOVA): none - >0.1; ^#^0.05–0.1; **0.001–0.01.

The interaction between species diversity and soil depth heterogeneity was observed only for legumes growth. In the low diversity treatment, legumes accumulated more aboveground mass in Hete-16 treatment than that in other soils; whereas in the high diversity treatment, legumes produced less aboveground mass in Hete-64 treatment compared to others (Fig. 2C). The interaction between diversity and soil patch had a marginal effect on legumes growth with legumes in low diversity communities producing more aboveground mass in deeper soil patches (60 cm) of Hete-16 treatment, while those in high diversity communities accumulated less aboveground mass in deeper soil patches of both heterogeneous soils (Fig. 3C).

**Figure 4:**
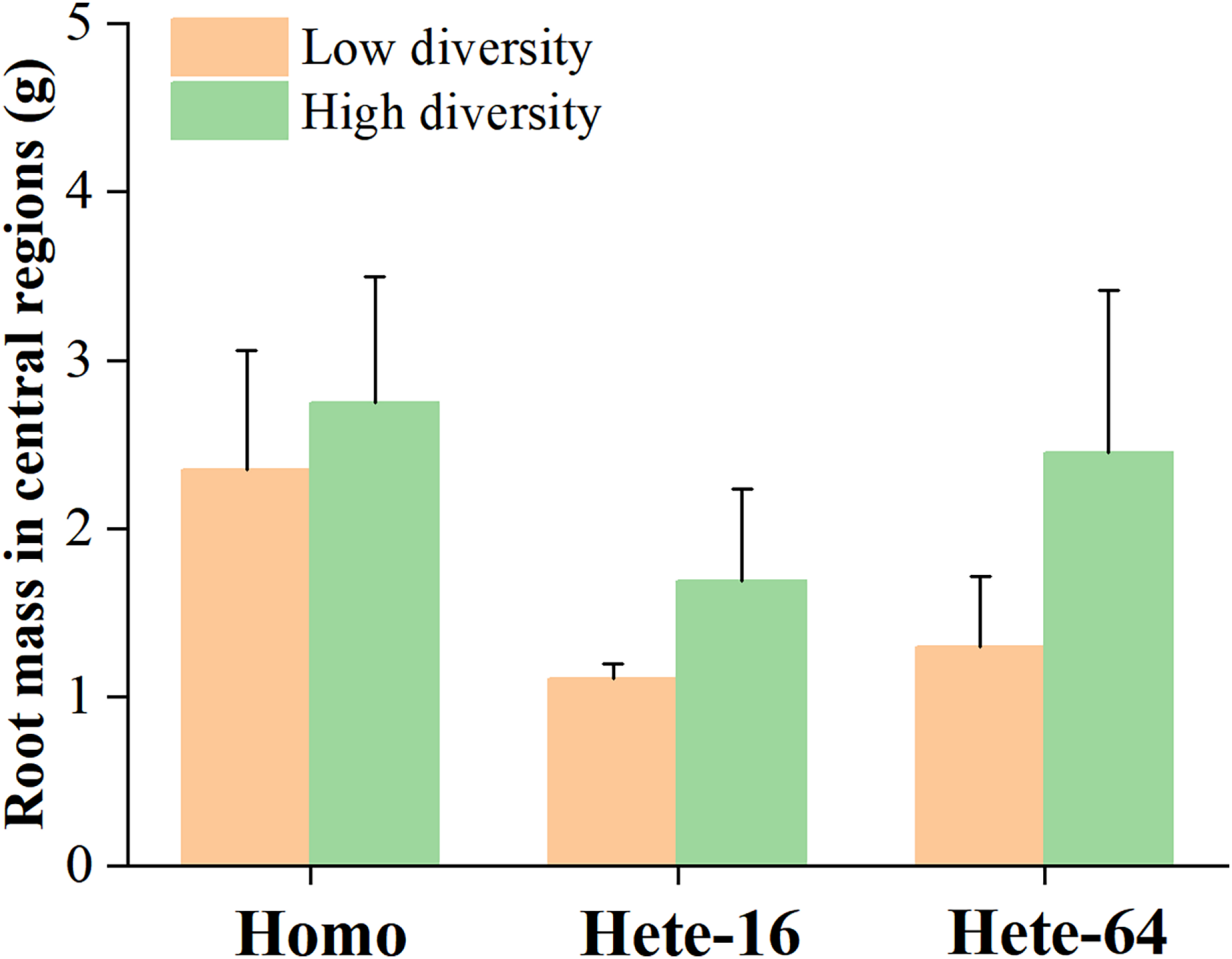
Effects of soil heterogeneity and species diversity on roots mass of community in central regions. The error bars are expressed as mean + SE. Symbols above pairs of bars show *P* (linear contrast based on ANOVA): none –>0.1.

**Figure 5:**
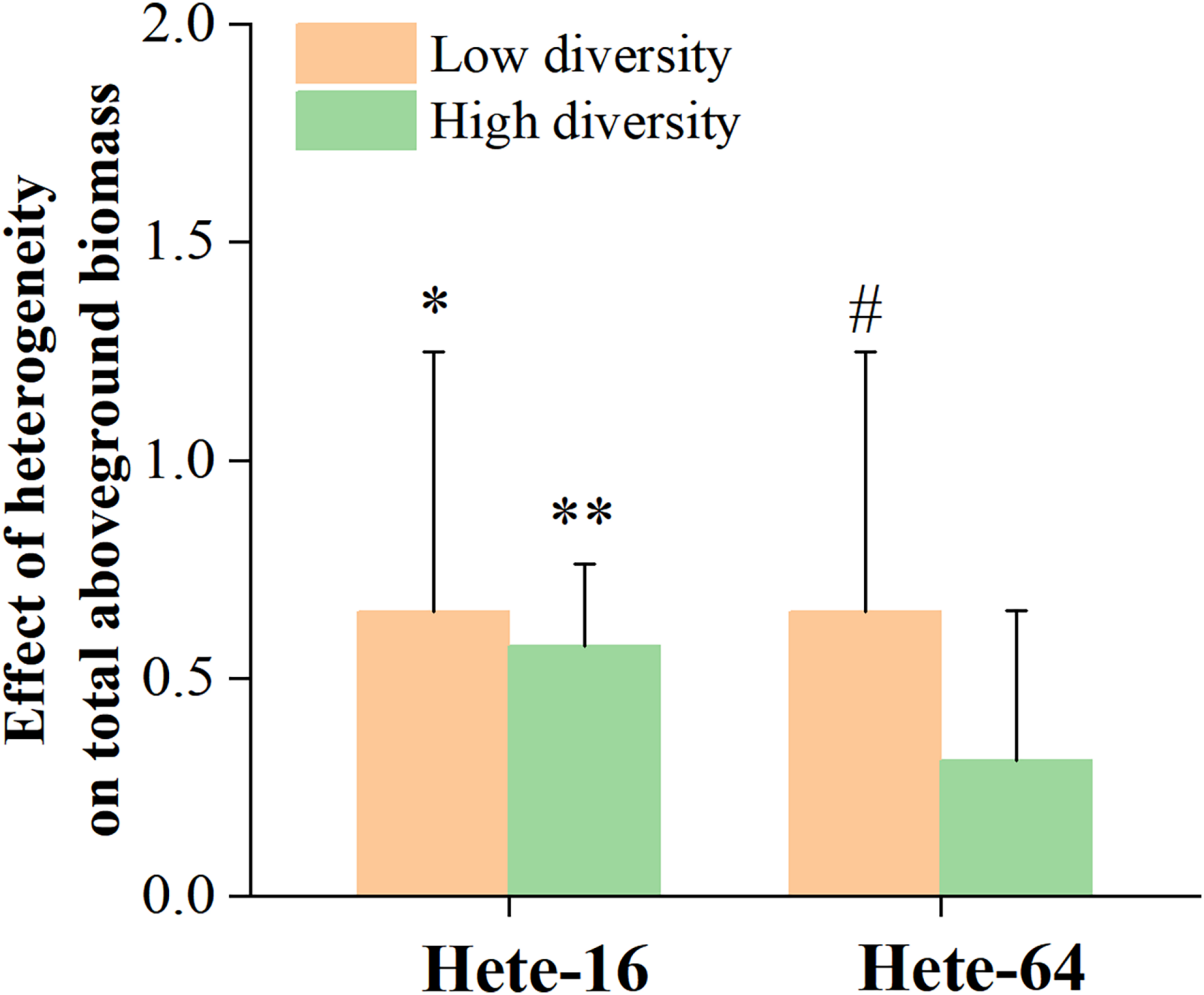
Differences between large patch and small patch heterogeneity with high or low diversity in total aboveground bio mass of community. The error bars are expressed as mean + SE. See texts for definitions of differences between effect of soil heterogeneity at two scales. Symbols abovebars show *P*: none - > 0.1; ^#^0.05–0.1; *0.01–0.05; **0.001–0.01.

Although the interaction between diversity and soil depth heterogeneity on forbs growth was not significant in ANOVA tables (Table 3), *post hoc* contrasts between individual means indicated that forbs in the high diversity treatment produced more aboveground mass in Hete-16 treatment by occupying deeper soil patches (Figs. 2D, 3D). No significant interaction effects were detected for the aboveground mass of grasses. Root mass in the central regions of the community was significantly influenced by soil depth heterogeneity (F = 10.481, *p* ≤ 0.001), with community under Hete-16 treatment producing less root mass than other treatments, particularly in low diversity treatment (Fig. 4). The effect of soil heterogeneity on total aboveground biomass of communities varied across two scales. Community under Hete-16 treatment produced more aboveground biomass across all diversity treatments, whereas under Hete-64 treatment, community tended to accumulate more aboveground biomass solely in low diversity treatment (Fig. 5).

## Discussion

The results support hypothesis that plant functional groups differ in their responses to soil depth heterogeneity. Legumes and forbs showed more positive responses to soil depth variation than grasses. This differential response among functional groups likely reflects inherent differences in root traits, particularly root system architecure (Mommer et al., 2012; Wijesinghe et al., 2001). Deep-rooted plants, including trees and perennial herbaceous species, are characterized by thick and elongated roots with low specific root length and strong penetration capacity. Shallow-rooted plants, such as annual herbaceous species, mosses, and shallow-rooted shrubs, possess fine and short roots with high specific root length and extensive branching (Freschet et al., 2021b). In this study, grasses typically have fibrous roots and stoloniferous rhizomes that primarily acquire nutrients horizontally and rarely to extend beyond 20 cm in depth. Given that soil depths in the experiment were 20, 40, and 60 cm, grass roots unlikely to reach deeper soil layers, resulting in limited responsiveness to soil depth variation. In comparison, *A. laxmannii*, a representative legume, possesses a deep taproot capable of accessing resources in deeper soil layers. *C. intybus* and *S. oleraceus* have roots exceeding 40 cm in length, showing a sensitive response to soil depth variation. These differences in root lengths therefore determined the capacity of plants to explore and acquire resources from deeper soil, particularly under heterogeneous conditions (Braun et al., 2022; Liu & van Kleunen, 2019).

A key aspect of this experiment was the integration of soil heterogeneity and vertical depth to examine the effects of soil depth variation on plant communities. The results support hypothesis that soil depth heterogeneity can enhances community productivity (Baer et al., 2019). Within the experimental communities, forbs contributed the largest proportion of total biomass, followed by legumes and grasses. Species such as *A. laxmannii*, *C. intybus*, and *M. sativa*, which are highly sensitive to soil depth and relatively large in size, directly enhanced the community’s response to such heterogeneity, particularly as their proportional contribution increased. An experiment in temperate grassland also suggested that increased soil depth substantially promoted forb abundance, hence enhancing species diversity and community aboveground mass (Dornbush & Wilsey, 2010). The results further indicate that species composition indirectly regulates community-level productivity responses to environmental heterogeneity, extending beyond nutrient distribution to variation in soil depth (Baer et al., 2019; Stevens & Jones, 2006).

The results also support hypothesis that soil depth heterogeneity can alter interspecific relationships among various plant functional groups. In natural grassland, legumes frequently emerge as the dominant species within the community due to their symbiotic rhizobia and growth characteristics (Bittlingmaier et al., 2026; Braun et al., 2022; Daleo et al., 2023). However, the performance of legumes in this study likely reflects the relatively weak competitive ability against forbs. Aboveground competition for light availability is asymmetric, with larger individuals usually having a competitive advantage (D’Andrea et al., 2024; Shen et al., 2024). *C. intybus* or *S. oleraceus* formed a relatively dense canopies with expanded leaves, reducing light availability for legumes and grasses. Plants located at container edges experienced less shading than those in the central positions. Therefore, legumes suffered more severe light limitation than grasses without extensive rhizomes and thin leaves.

An interesting result was that legumes exhibited contrasting responses to soil depth depending on community diversity. This shifts in root strategy may be attributed to belowground niche competition: legumes avoid or escape favorable deeper soil layers to reduce competitive inhibition from forbs (particularly Compositae), a process consistent with competitive exclusion (Orrock et al., 2015; Petry et al., 2018). In the absence of stronger competitors such as Compositae, legumes can effectively occupy their preferred deeper soil layers. This differs from previous studies, which suggested that enhanced soil depth provides vertical resources niche that facilitate mutual coexistence among species (Hou et al., 2023; Van Steijn et al., 2025). Our results found that soil depth heterogeneity intensified interspecific competition between legumes and forbs, compelling legumes to alter their root preference. Results of root mass in central regions further proofed this intensified interspecific competition, representing less allocation to belowground mass rather than to aboveground mass. Unlike asymmetric aboveground competition for light, belowground competition for nutrients and space is mostly symmetric (D’Andrea et al., 2024; DeMalach et al., 2016). Consequently, variations in root length and architecture may enables differential utilization of belowground space, reducing niche overlap (Freschet et al., 2021a; Martorell et al., 2015).

Although increased species diversity reduced legumes growth, it still significantly enhanced community productivity. This outcome may reflect selection effects among component species induced by higher richness (Zuppinger-Dingley et al., 2014). Numerous studies indicate that higher species richness leads to greater biomass output (Gross et al., 2014; van der Putten, 2017). The richness in high diversity treatment was twice that of the low diversity treatment, allowing forbs directly to contribute the highest total aboveground mass across all treatments. Based on its higher proportional contribution to aboveground mass and larger individuals size, *C. intybus* is logically inferred to be a dominant species that strongly influences aboveground mass accumulation and the community’s response to soil heterogeneity. A meta-analysis on plant diversity effect on productivity also point that selection effects are evident in container experiments but less significant in forests and wetlands, primarily due to marked differences in monocultures productivity among species (Chen, Xiao & Chen, 2025). Greater variation in species productivity leads to more pronounced selection effects.

The effect of small patch heterogeneity on plant growth resembled that of homogeneous treatment, reducing only legumes growth. One possible explanation is that the patch size in the Hete-64 treatment (6.25 cm × 6.25 cm) may have been too small, potentially below the scale of individuals growth. Relationships between soil heterogeneity and species diversity are highly dependent on the spatial scale at which diversity is quantified (Havrilla & Villarreal, 2024; Tamme et al., 2010). Plants with greater root foraging ability can easily cross multiple soil patch boundaries, rendering heterogeneity ineffective or functionally irrelevant (Liu et al., 2020a). Comparatively, species with smaller foraging scales show higher foraging precision, while those with larger foraging scales tend to forage less precisely (de Kroon et al., 2009). Furthermore, the vertical nature of soil depth heterogeneity differs from horizontal nutrient heterogeneity (vertical *vs*. horizontal), suggesting that the small-scales vertical heterogeneity poses greater challenges for effective root foraging. This phenomenon mirrors natural systems, where soils with higher stones density often support sparser vegetation. Thus, soil heterogeneity at small spatial scales may become a form of habitat fragmentation (Einsmann et al., 1999; Roiloa & Retuerto, 2006).

## Conclusions

Soil depth heterogeneity enhances community productivity primarily by promoting the growth of legumes and forbs, whereas grasses remain largely unresponsive. Species diversity influences community assembly by inducing a selection effect in forbs, increasing their proportional contribution to aboveground biomass and strengthening interspecific competition under heterogeneous soil conditions. Legumes adopt a flexible root growth strategy, avoiding deeper soil layers to mitigate competitive pressure. Seed germination rates and competitive exclusion contribute to shifts in community composition, increasing variability and complicating species-level interpretation. Future studies using transplanted seedlings or long-term field experiments are required to better elucidate community dynamics and the biotic and abiotic mechanisms governing species coexistence across gradients of diversity and soil heterogeneity.

## Supplemental Information

10.7717/peerj.21225/supp-1 Supplemental Information 1 Original data of plant growth. Sheet 1 and 2 are used for repeated-measures-ANOVA, while sheet 3 and 4 are used for ANOVA to examine the former results. Three-way, repeated-measure ANOVAs were used to evaluate the effects of soil heterogeneity (heterogeneous or homogeneous), diversity (high or low), and soil patch (shallow or deep, repeated measure) on total aboveground bio mass, aboveground mass of grasses, aboveground mass of legumes, aboveground mass of forbs, and root mass. Heterogeneity and diversity were treated as fixed effects, block (i . e. species composition) as random effect, and soil patch as a repeated measure. Soil patch was a non-independent within-subjects variable nested within heterogeneity.

10.7717/peerj.21225/supp-2 Supplemental Information 2 Comparison of root architecure of *Cichorium intybus*. *Cichorium intybus* in two soil depth shows distinguished root architec ure

10.7717/peerj.21225/supp-3 Supplemental Information 3 Root length of *Cichorium intybus*. The root length of *Cichorium intybus* in deeper soil is approximately 60 cm.

## Additional Information and Declarations

### Data Availability

The following information was supplied regarding data availability:

The raw data is available in the Supplemental File.
