# Supplementary figures and images for: Combined effects of species diversity and soil depth heterogeneity on plant functional groups and community productivity
Source: PeerJ. 2026 May 1;14:e21225. doi: 10.7717/peerj.21225 (PMC13138296; doi:10.7717/peerj.21225)

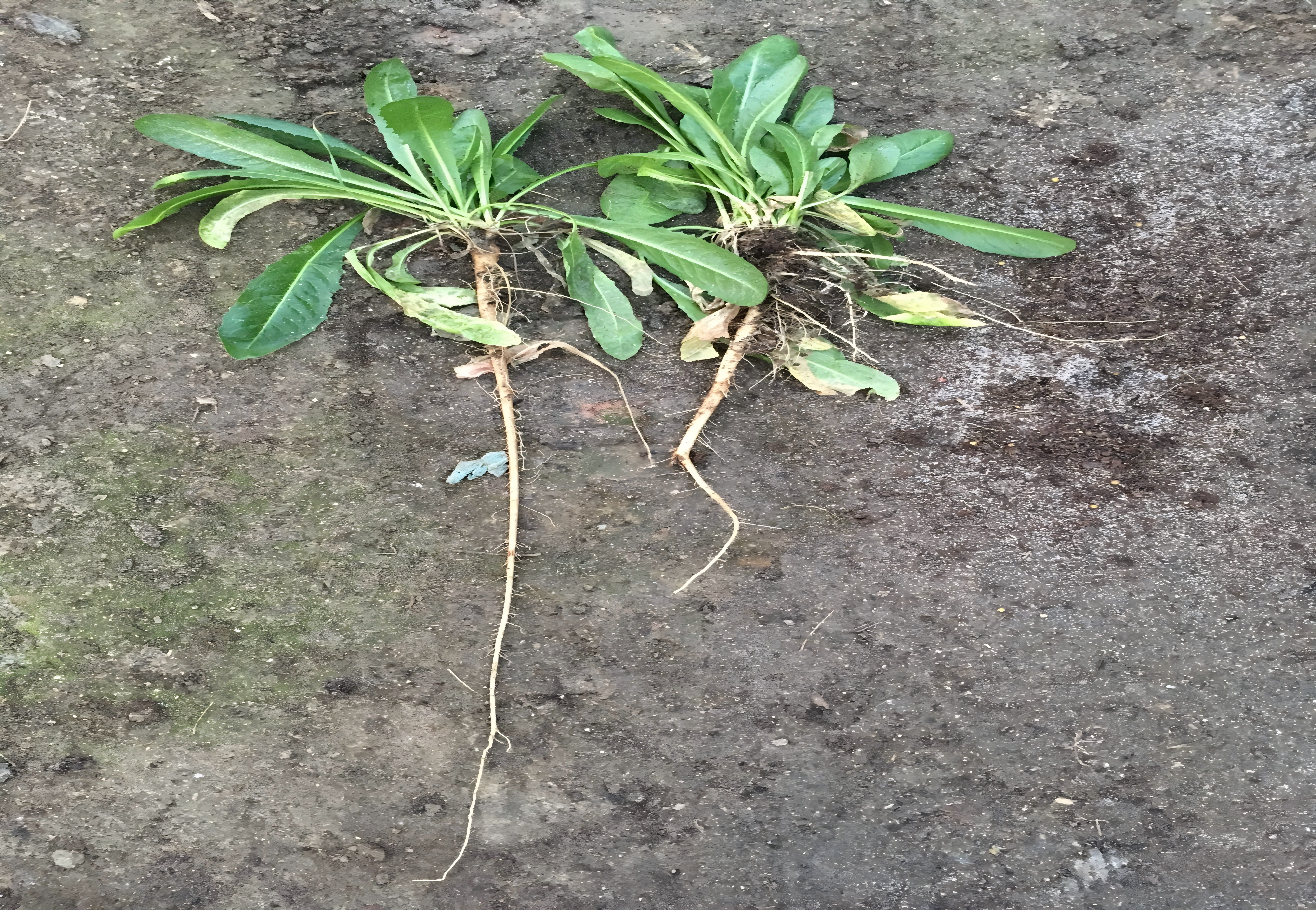

Supplement: Supplemental Information 2 — Cichorium intybus in two soil depth shows distinguished root architec ure [file peerj-14-21225-s002.jpg]

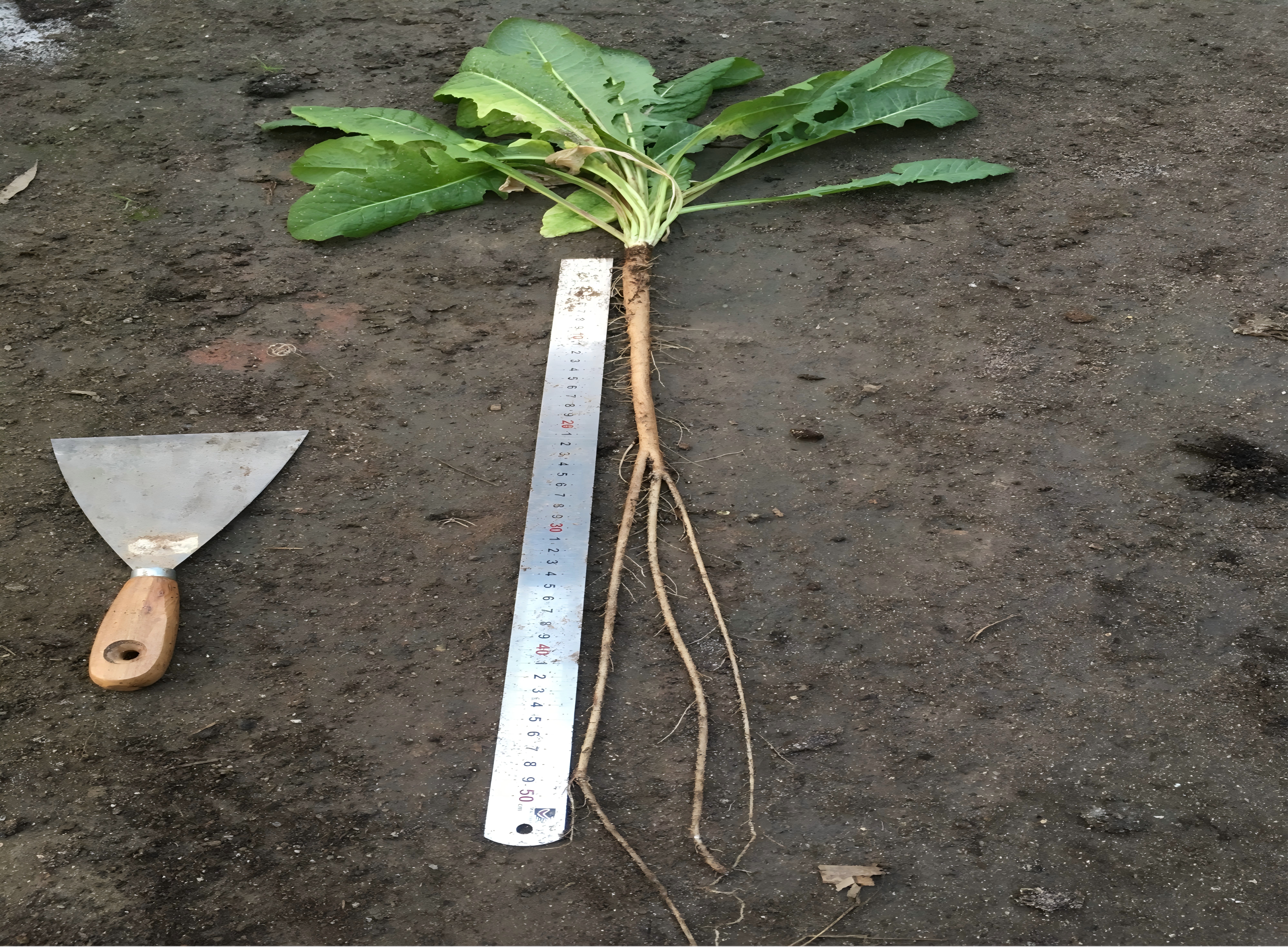

Supplement: Supplemental Information 3 — The root length of Cichorium intybus in deeper soil is approximately 60 cm. [file peerj-14-21225-s003.jpg]
